# A New Association Scheme for Handling Node Mobility in Cluster-Tree Wireless Sensor Networks

**DOI:** 10.3390/s20195694

**Published:** 2020-10-06

**Authors:** Rogério Casagrande, Ricardo Moraes, Carlos Montez, Francisco Vasques, Erico Leão

**Affiliations:** 1 Department of Computer Science, University of the Far South of Santa Catarina, 88806-000 Criciúma, Brazil; roc@unesc.net; 2 Department of Automation and Systems, Federal University of Santa Catarina, 88040-900 Florianópolis, Brazil; carlos.montez@ufsc.br; 3 Department of Computing, Federal University of Santa Catarina, 88905-120 Araranguá, Brazil; ricardo.moraes@ufsc.br; 4 INEGI, Faculty of Engineering, University of Porto, 4200-465 Porto, Portugal; 5 Department of Computing, Federal University of Piauí, 64049-550 Teresina, Brazil; ericoleao@ufpi.edu.br

**Keywords:** IEEE 802.15.4, handover, node-mobility, health monitoring, cluster-tree

## Abstract

Node mobility in multi-hop communication environments is an important feature of Wireless Sensor Network (WSN)-based monitoring systems. It allows nodes to have freedom of movement, without being restricted to a single-hop communication range. In IEEE 802.15.4 WSNs, nodes are only able to transfer data messages after completing a connection with a coordinator through an association mechanism. Within this context, a handover procedure needs to be executed by a mobile node whenever there is a disconnection from a coordinator and the establishment of a connection to another one. Many applications, such as those found in health monitoring systems, strongly need support for node mobility without loss of data during the handover. However, it has been observed that the time required to execute the handover procedure is one of the main reasons why IEEE 802.15.4 cannot fully support mobility. This paper proposes an improvement to this procedure using a set of combined strategies, such as anticipation of both the handover mechanism and the scan phase enhancement. Simulations show that it is possible to reduce latency during the association and re-association processes, making it feasible to develop WSN-based distributed monitoring systems with mobile nodes and stringent time constraints.

## 1. Introduction

A Wireless Sensor Network (WSN) may be composed of a large number of low-cost devices distributed within a particular area of interest. Over the last few years, WSNs have been used for agriculture, environment, industry and health monitoring purposes [1]. IEEE 802.15.4 standard [2] defines one of the most widely used protocols for deploying WSNs, specifying the physical layer and medium access control sublayer for low-data-rate wireless connectivity. An IEEE 802.15.4 network operates either in one of two topologies, depending on application requirements: star topology or peer-to-peer topology. A cluster-tree topology is a special peer-to-peer topology that has been shown to be one of the most suitable for deploying wide-scale WSNs [3]. It is noteworthy that, regardless of the topology used, every node in an IEEE 802.15.4 network must be associated with the network, through an association procedure, before it can exchange messages (for more information about association and re-association process of the IEEE 802.15.4, please refer to [2]).

Many WSN monitoring applications require mobility of sensor nodes; therefore, enabling node mobility has become a significant challenge in WSN research [4,5,6]. Many wireless standards and technologies do not efficiently support node mobility [7], impairing their use, especially if WSNs are being used for supporting critical applications with real-time constraints and reliability requirements.

Ideally, mobility implies that devices moving along the monitored area do not suffer connection losses, requiring the use of appropriate association/re-association mechanisms. This means that nodes must decide at appropriate time instants to disconnect from their coordinators in order to associate with another with a better signal, via a mechanism known as a handover. As mobility support involves several relevant issues, such as the best moment to start the handover procedure and the time required to complete this operation [8], various design issues must be carefully considered, such as adequate beacon scheduling [3], node speed upper bounds and direction [9,10,11], and time spent in the scanning phase [9,12]. However, regarding the time and energy costs of the handover procedure, the most critical aspect is the association algorithm due to the scan phases and the Media Access Control (MAC) involved [13].

This paper aims to improve mobility support for WSN nodes, acting upon the association and handover procedures. These improvements aim to reduce the time interval during which a Mobile Node (MN) remains out of communication with the network. We consider an existing WSN monitoring communication infrastructure organized as a cluster-tree beacon-enabled topology. We also consider that one or more mobile nodes are able to join to the network, at any instant, in order to transmit messages with real-time requirements. In this context, there are several issues that must be taken into account. For example, how to integrate mobile source nodes into a pre-established cluster-tree network (association and re-association phases); how to guarantee connectivity without packet losses (handover, buffers); and how to guarantee timing requirements (resource allocation).

This proposal is based on a set of distinct approaches, and its main contributions can be summarized as follows: (1) we present a new handover mechanism compatible with the IEEE 802.15.4 standard where; (1.1) the handover decision is anticipated; (1.2) the orphan scan step is suppressed; (1.3) the number of channels used in the scan operation is minimized; (1.4) the message exchange sequence defined in the IEEE 802.15.4e optional FastA association mechanism is used; (1.5) the proposed handover mechanism has been assessed by simulation and the achieved results highlight significant improvements in the whole handover procedure; (2) a suitable setup of the IEEE 802.15.4 MAC layer parameters is proposed for the association procedure; and (3) a review of state-of-the-art approaches addressing improvements in the handover process of the IEEE 802.15.4 standard.

This paper is organized as follows. Section 2 discusses related works, presenting a systematic literature review about handover mechanisms in wireless sensor networks. Section 3 describes the assumed network model, the problem statement and the proposed handover scheme. Section 4 gives an assessment of the proposal via simulation. Finally, Section 5 presents conclusions and future works.

## 2. Related Works

Despite the importance of the association process in the IEEE 802.15.4 networks, most of the research related to this process focuses only on the analysis and the evaluation of its performance [14]. This section briefly describes relevant proposals found in the literature that somehow improves this process.

**Figure 1:**
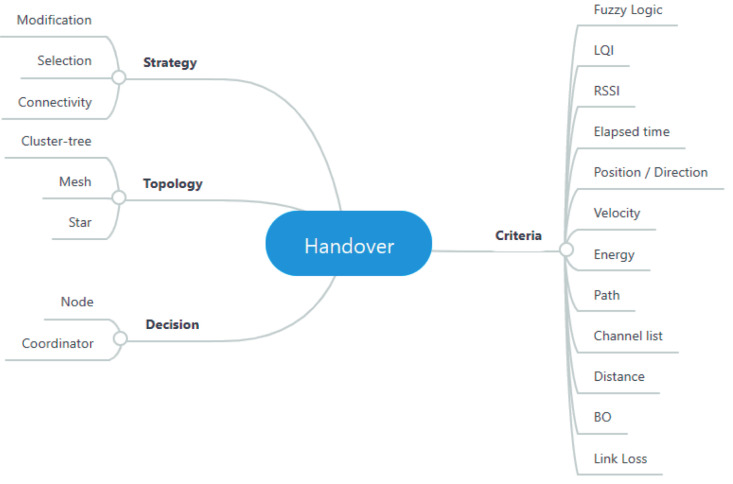
Improvements in the association process.

The research works reported in this section are organized and classified according to four axes: (i) strategy; (ii) criteria; (iii) topology; and (iv) decision. The first axis is used to classify the state-of-the-art proposals, and is related to how each proposal improves the association process in terms of three aspects: (a) modification to the association process, describing proposals where the message exchange process defined in the standard is modified; (b) coordinator selection, which involves an improvement in the selection of the coordinator point; and (c) connectivity, in which the duration of the association between a node and coordinator is addressed. These approaches are described, and then classified according to the other axes. The second classification axis identifies the criteria used to implement a particular strategy that is defined to improve the handover process. The third axis aims to identify the defined network topology, while the fourth is related to the handover decision, which can be initiated in the MN or the Access Point (AP). Figure 1 illustrates the proposed taxonomy.

### 2.1. Modification in the Association Process

The association process of IEEE 802.15.4 standard is defined in [2]. This subsection presents some relevant state-of-the-art proposals that modify this process.

In Zhang et al. [14] is proposed an association mechanism similar to FastA [2]. This scheme was called Simple Association Process (SAP). The main similarity is that the coordinator does not include the associating response in the list of pending transactions, i.e., the device just waits for the join response command sent by the coordinator, eliminating the need for request command data.

Meng and Han [9] proposed an association mechanism intended to support real-time applications. The Fast Association Mechanism (FAM) modified both the initialization and the channel scanning procedures of the standard association mechanism. In channel scanning, FAM stops a scanning process whenever a suitable Personal Area Network (PAN) has been found, reducing the time interval required to finish this process. Additionally, as in the previously cited proposal, FAM uses direct transmission to forward the join response command to the node.

In Shapit et al. [11], the authors present a new association scheme, in which coordinators transmit their beacon frames using a dedicated channel. This proposal allows a node to acquire network information from all coordinators in its vicinity by checking only one channel. They also proposed to use the Link Quality Indicator (LQI) as a means to anticipate a node’s loss of connectivity.

Yun et al. [15] reported that one of the main problems of association mechanisms is the delay caused by the full channel scanning process, that can reach 90% of the total handover interval and contributes to high packet loses and to the random loss of connection. Basically, they propose a new mechanism that eliminates the channel scanning delay, where the MN creates, and continually updates, a table to store the MAC address, the available channel number and the LQI of the neighbor coordinators. Then, whenever the LQI of the MN drops below a predefined threshold, the MN directly triggers the re-association instead of running a new scanning process.

Similarly to the previous work, Nepali and Shin [16] also presented an association technique to remove the full scanning process. They also proposed that the MN must maintain a list of operating channels for all the coordinators in the vicinity. Then, whenever the MN needs to find a new coordinator, it scans only those channels, reducing the number of channels to scan and, consequently, decreasing the total association time.

In Chong et al. [17], the authors proposed a set of algorithms to define the Contention Window (CW) parameter of the Carrier Sense Multiple Access with Collision Avoidance (CSMA-CA), according to the measured content level. A Markov chain is used to model the number of devices disassociated with a coordinator and the operation of the devices is modeled based on statistical concepts. These algorithms have been proposed to reduce the total association time.

### 2.2. Selecting Appropriate Coordinator

The IEEE 802.15.4 standard does not define criteria for selecting the coordinator, since these criteria vary according to the application needs. With the aim to save power and improve latency, some papers have proposed criteria for the selection of the most suitable coordinator.

Some criteria for optimizing the topology formation step of IEEE 802.15.4/ZigBee WSNs were proposed by Ouni and Ayoub [18]. These criteria are of two types: the first focused on single-hop topologies and the other on multi-hop topologies. Both types of criteria aim to balance the nodes’ energy consumption and message delays. A re-association method was also proposed, which assumes that the WSN topology can be dynamically changed, taking into account the nodes’ energy consumption over time.

Zinonos and Vassiliou [19] proposed a handoff scheme named the Burst Loss Algorithm, which considers the Received Signal Strength Indication (RSSI) and link loss when triggering the handoff procedure. This scheme initially uses an RSSI value threshold; whenever the RSSI is below this predefined threshold, the MN will start searching for a new AP. A hysteresis bound is then used for link loss: whenever the average link loss value is above a predefined value, the MN starts searching for another AP. In addition to this RSSI-based solution, the authors also propose a handover decision where mobile nodes must be associated with two links, the current and the new candidate. The quality of these links are constantly monitored, through the sending of burst probes, in order to use this information for the handover process.

Fotouhi et al. [20] propose a handover process based on a probabilistic model, where the RSSI, node speed, energy level, traffic load, number of hops to the AP and link quality metrics are evaluated. They demonstrate the impact of these parameters to decide when the handover should be triggered. Results show a significant improvement in the mobility support of low-power networks.

Yazdi et al. [21] assessed the performance of the IEEE 802.15.4, considering a scenario composed of static APs and MNs operating at different speeds. Their preliminary results showed a need to improve the handover procedure. Then, three different decision schemes to start a handover process were proposed: blind, predictive, and threshold-enabled predictive (TEP) schemes. The blind scheme allows nodes to select the AP based only on the RSSI values. In the Predictive and TEP schemes, the MN estimates its distance from the APs and the movement direction through the RSSI values, selecting the appropriate AP to associate.

Ayoub and Ouni [22] have proposed a new re-association approach for WSN to guarantee the connectivity of MNs through a reliable handover mechanism. The main idea of this work is to choose an adequate coordinator for each one of mobile nodes in order to obtain appropriate child-parent relations and optimized network topologies. The handover process can be triggered by both the coordinator node (based on its remaining residual energy) and the MN (based on location change). Simulation results showed that the proposed scheme saves energy consumption and improves the packet delivery ratio and network throughput. In [23], the same authors propose a procedure, where the MN anticipates the handover whenever it detects an LQI degradation. It then performs a fast re-association based on a ‘smart’ criterion. This criterion is given by the sum of the inverse of the remaining energy of the coordinators in the path to the sink node and allows the selection of the shortest path with the maximum remaining energy power, thus optimizing power consumption and decreasing latency.

Kaur et al. [24] classify the handover mechanisms according to how the process has been initiated, that is, by the coordinator or MN. When the process starts in the MN, whenever it moves away from the coordinator’s coverage area, it starts a search for another candidate coordinator. The selection of the coordinators is based on the residual energy of the coordinator and the LQI. When the process is based on the coordinator, the node initiates the handover process whenever it verifies that the power of its coordinator is lower than a defined threshold. The remaining energy in the coordinator is periodically checked. When it is below the threshold, the coordinator sends a disassociation command to its nodes and the handover is performed.

Caldeira et al. [25] design a handover mechanism for IEEE 802.15.4 operating in beacon-disabled mode. The idea is that the APs can search for a specific MN instead of continuously monitoring the RSSI between the node and the corresponding AP. This mechanism is assessed considering an infirmary scenario, where patients have a set of sensor nodes attached to their bodies and can freely walk in this area.

Filho et al. [26] propose a fuzzy-based decision scheme to be used in the handover procedure of IEEE 802.15.4 standard operating in beacon-enabled mode. This scheme, named Group-Weighted Fuzzy Quantitative Decision Algorithm (group-weighted FQDA), assembles parameters (battery size, signal strength, etc) into groups (energy, link quality, etc) according to their proximity, defining profiles according to the type of the application. The group-weighted FQDA was assessed, and showed best performance when compared with other solutions. This algorithm can be extended to a large number of metrics and does not require computational or storage capabilities, thus allowing good adaptability to the problem of WSN mobility.

### 2.3. Improving Connectivity between MN and AP

Hussain and Pyun [8] investigated the question of coordinator discovery and association for mobile end nodes, which need to perform these operations due to these frequent cluster changes. The authors propose a scheme named Coordinator-Assisted Passive Discovery and Association for mobile end devices, which is intended to provide rapid discovery of coordinators and to reduce the association time during a cluster change. In this scheme, a parent endpoint coordinator uses LQI to check if any mobile node is moving away from its cluster. If so, the coordinator asks the neighboring coordinators to assign a temporary network address to the mobile device. This action reduces the association time by eliminating timeout responses when exchanging messages in the association procedure.

In another work, the same authors [27] propose a modification of the IEEE 802.15.4 re-association procedure that can decrease the frequency of re-association and latency for a beacon-enabled WSN with cluster-tree topology and mobile nodes. The scheme named Preemptive Re-association was designed to preventively detect loss of connectivity. In addition, the method performs a fast re-association procedure and selects coordinators that can provide increased connectivity during re-association. In the case of networks with a high beacon order parameter, the method also attempts to decrease the beacon interval of coordinators that are close to the mobile devices. In short, a make-before-break approach is first used to reduce the delay in connectivity loss detection after cluster switching. The orphan scan is not used, since in this approach the MN is connected to the AP when scanning, thus avoiding becoming an orphaned node. Passive scanning is performed only on the current channel to decrease the scan time. Finally, the Beacon Order (BO) parameter of neighboring coordinators is decreased when the parent coordinator perceives weak connectivity with any of its child nodes. This helps a child device with low connectivity to quickly select a new coordinator. To reduce the handover frequency, a new criterion for coordinator selection is proposed that takes into account the LQI between the mobile node and the coordinator. As a result, connectivity can be improved by selecting the coordinator with the best signal quality.

In Postigo-Malaga et al. [28], both a WSN architecture and a scheme for tracking mobile nodes in outdoor scenarios are proposed. The architecture assumes a ZigBee network, where there is always an intersection in the coverage areas of neighboring AP nodes. According to the authors, there is no need for a handover process because all the AP nodes are configured with a unique PAN identifier, and the AP with higher RSSI value will receive the messages from mobile nodes and send them towards the coordinator. The architecture was assessed in a real urban environment where AP nodes were installed at semaphores, and communicate with MNs installed in vehicles. The scheme showed promising results in most of the cases. However, it would be interesting to have further results for a larger number of nodes to assess scalability problems.

### 2.4. Synthesis of the State-of-the-Art

**Figure 2:**
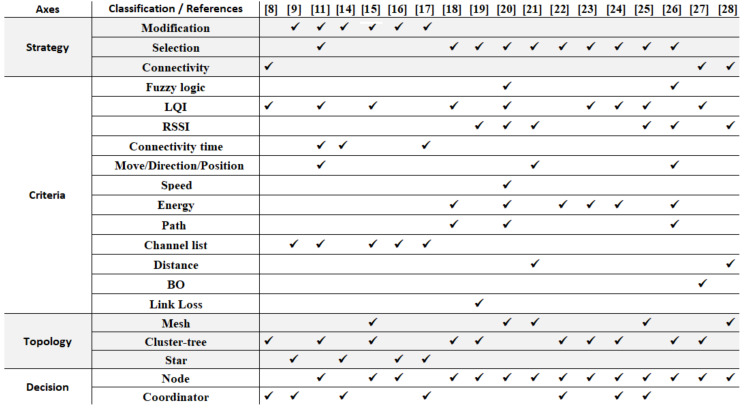
Synthesis of the reported proposals.

In the previous subsections, some of the most relevant IEEE 802.15.4 handover proposals were presented according to the main strategy used to improve the handover process. In this section, these proposals are classified according to the remaining axes. Figure 2 classifies the reported proposals according to the four classification axes previously defined.

Regarding the strategy adopted for handover, most of the above studies anticipate the selection of a more appropriate coordinator when degradation of the connection between the MN and the coordinator is detected [8,11,18,19,20,21,22,23,24,25,26]. Another relevant characteristic is that one of the main modifications to the association mechanism is to reduce the number of scanned channels in the case of loss of connectivity [9,11,15,16,17], while some other schemes try to reduce the association time.

In terms of the metrics to select a suitable time instant to perform the handover, it can be seen from Figure 2 that the vast majority of the studies are based on RSSI [19,20,21,25,26,28] and/or LQI [8,11,15,18,20,23,24,25,27] for handover decision-making. Other works use the residual energy of the mobile nodes as a criterion [18,20,22,23,24,26]. Some other studies that use the direction of movement or position of the mobile node as a decision criterion can also be found [11,21,26]. Finally, refs. [21,28] consider the distance from the mobile node to the coordinator and others [18,20,26] the path from the mobile node to the coordinator. Some of these proposals are based on multiple criteria [11,18,20,26], and the remainder considers only one, or at most two, criteria to select the best instant for the handover decision.

It is clear that the cluster-tree topology was predominantly selected, followed by a mesh topology. This is an expected result, as mesh and cluster-tree topologies make it possible to cover larger areas. Regarding whether the decision on the handover is taken by the mobile node or the coordinator, it was observed that in a large number of works is the mobile node that initiates this process. Some other schemes propose an initiation process involving the coordinator node [8,9,14,17]. Several studies were also found in which decision making was distributed between the MN and the coordinator [22,24,25].

Through a systematic review of the literature, this section raised state-of-the-art proposals for improving the IEEE 802.15.4 standard association mechanism, providing the theoretical basis necessary to improve the handover process. This study showed that it is necessary to take a step forward in the proposals for IEEE 802.15.4 association mechanisms.

## 3. System and Network Model

Recently, WSN are also being used to support monitoring applications in health, agriculture, industrial and smart cities domains [1]. Regarding the health domain, also called Internet of Medical Things (IoMT), sensor monitoring applications are proposed for a wide range of applications, but they bring many challenges concerning reliable and timely delivery of critical signals [29]. For example, heart rate data have stringent time constraints, and one of the major challenges in this context is to guarantee the quality of service for the data transfer of from several sensors that have different priorities, where a delay could have serious consequences for patients [30].

This paper addresses, as an application example, a research issue that has been found in a real health monitoring system involving a Body Area Network (BAN). The proposed architecture is intended to monitor a set of mobile nodes, which are communicating through a set of infrastructure nodes that form a multi-hop network. This multi-hop architecture, adapted from [31], is composed of three layers: Intra-BAN, Inter-BAN and Beyond-BAN. The Inter-BAN layer is based on the node backbone concept, serving as a communication infrastructure for one or more MNs, localized in the Intra-BAN layers, to transfer their data towards the monitoring system in the Beyond-BAN layer. This last layer is formed by network gateways and cloud services. It is important to highlight that more recent works, e.g., [29], also define similar architectures for individual and group health monitoring systems, where one of the many issues is to guarantee the message transmission from MNs to the gateway.

Basically, the main target of this paper is to improve the quality of the communication line between MNs and the Inter-BAN layer, guaranteeing reliable monitoring communication from the patient till the beyond-BAN layer. Within this context, this paper contributes to the association mechanism of WSNs structured in cluster-tree topology. The main contributions can be divided into two main parts: network infrastructure and handover mechanism.

### 3.1. Network Infrastructure

As the defined architecture must be able to support mobile nodes and a large coverage area, we assume a WSN-based communication infrastructure with a cluster-tree topology for the Inter-BAN layer. These assumptions require a specific look to, at least, the following technical challenges: the possibility of the collision of beacons; the integration of mobile nodes in a pre-established network (association and re-association); the guarantee of connectivity without packet losses (handover, buffers); and the guarantee of time requirements through the efficient allocation of communication resources (superframe duration).

#### 3.1.1. Network Scheduling

**Figure 3:**
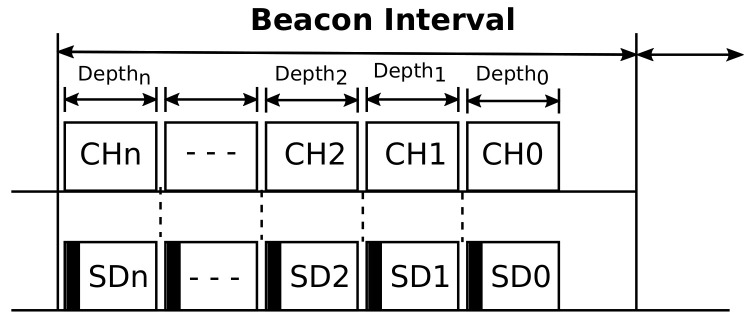
Bottom-up cluster active period scheduling.

In cluster-tree networks, clustering is just a logical grouping of nodes. When two physically close clusters transmit beacons at similar time instants, data transmissions from adjacent clusters will probably collide, causing retransmissions and packet losses. In order to avoid such inter-cluster collisions, we consider the use of a beacon scheduling algorithm based on a previous work [3], preventing overlaps of beacon frames from adjacent clusters. Thus, the cluster scheduling is organized following a bottom-up scheme, where superframe durations (i.e., the time interval during which the cluster may remain active) are ordered in a bottom-up sequence, i.e., first the deepest clusters, then clusters of the next lowest depth, and so on, until reaching the PAN coordinator (CH0), as illustrated in Figure 3. Considering that active periods of the clusters are composed only of Contention Access Periods, any node within the cluster can try to send its messages during the active period of its cluster.

To implement this beacon-scheduling scheme, the following protocol constraints must be respected: the length of Beacon Interval (BI); buffer sizing and node speed.

#### 3.1.2. Length of BI

The length of the BI must ensure that all superframe durations ($SD_{j}$) can be scheduled during the BI. Therefore:

(1) $$BI\geq \sum_{j=1}^{N_{CH}}SD_{j}$$

where *BI* is the beacon interval, *SD* is the superframe duration and $N_{CH}$ is the number of Cluster-Heads (CHs). Nevertheless, higher BIs will highly increase the end-to-end communication delays. Thus, the BI should be carefully defined in order to prevent high end-to-end communication delays.

Considering that a specific sensor node *i* is able to send messages during its cluster active period, and also that it generates a new message every period $P_{i}$, the message periodicity of each node imposes a restriction upon the BI. However, as the message generation period of a MN is not synchronized with the beacon period, a new message may be generated after its active period. In that case, it will be sent just during the next cluster active period.

Within this context, in order to guarantee that a message may be transferred before the next message generation, $P_{i}$ must always be larger than the BI plus δ, where δ is the maximum time required to transmit a message. Then, the following constraint must be satisfied:

Then:

(2) BI≤min{P}−δ,

From Equations (1) and (2), it follows that:

(3) $$\sum_{j=1}^{N_{CH}}SD_{j}\leq BI\leq min\left\{P\right\}-\delta$$

Therefore, Equation (3) represents a protocol constraint that must be guaranteed when setting up cluster-tree networks.

#### 3.1.3. Buffers Sizing

Each $CH_{j}$ must also store, in the worst case, the entire set of data messages generated from all messages streams of its child nodes, including from its connected mobile nodes. That is, there is a following buffer constraint:

(4) $$B_{j}\geq \sum_{i\in S_{below}^{j}}S_{n},\;\mathrm{for}\;1\leq n\leq N_{nodes}$$

where $B_{j}$ is the MAC buffer size of the $CH_{j}$, and *n* corresponds to the number of generated messages by $S_{below}^{j}$ message streams located in the child nodes during one BI.

The mobile node must also be able to store the messages generated from its own message streams during a handover period, i.e., during the time interval required to complete the entire re-association procedure. Then the buffer sizing of each node is given by:

(5) $$B_{MN}\geq \sum_{i\in S}\left\lceil \frac{P_{i}}{t_{handover}}\right\rceil$$

where $B_{MN}$ is the MAC buffer size of the MN, $P_{i}$ is the period of message stream *i*, and $t_{handover}$ is the time taken to complete the re-association process. As $B_{MN}$ must be an integer number, $\left\lceil x\right\rceil$ represents the ceiling operator, that is, the operator that rounds up the real number *x* into to the nearest integer number.

#### 3.1.4. Node Speed

Node speed is another important issue to consider for the handover procedure. If the mobile node moves faster, the time to perform handover must be shorter. On the other hand, if it moves more slowly, there is more time to complete this procedure. This often creates problems for a moving node because it will receive a beacon from a coordinator and try to associate but may not be able to finish the association process on time.

**Figure 4:**
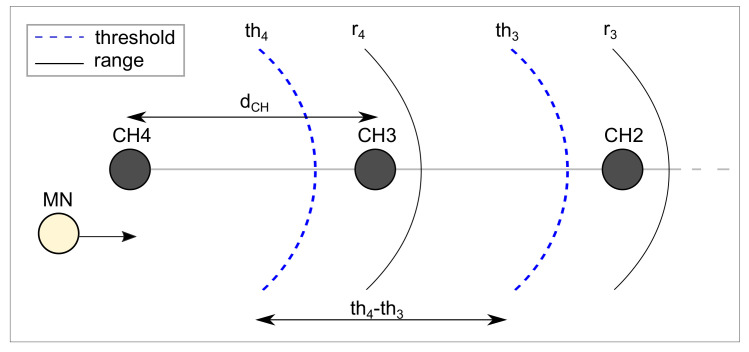
Node speed analysis.

Figure 4 illustrates how the node speed relates to the handover. It considers an operating range (*r*) and a threshold (th) for each cluster-head. A threshold $th_{j}$ is defined as the last time instant when a mobile node *j* must start a re-association procedure in order to avoid message losses. In the example, MN is initially associated with CH4 and moves toward CH3. When it reaches CH4 threshold ($th_{4}$), it starts a re-association procedure leading to the association to CH3. The handover must be complete before MN reaches CH3 threshold ($th_{3}$). If the device moves too fast, while waiting for the response from CH3, it may already be out-of-range from CH3. Assuming identical and symmetrically distributed CHs, the distance between thresholds ($th_{4}-th_{3}$) may be considered the same distance as between CHs ($d_{CH}$). Therefore, the maximum time to complete the handover procedure is given by:

(6) $$t_{handover}\leq \frac{d_{CH}}{V_{MN}}$$

where $t_{handover}$ is the time to complete the handover procedure, $d_{CH}$ is the distance between cluster-heads and $V_{MN}$ is the speed of the mobile node.

If the MN moves faster, the threshold $th_{j}$ must be defined closer to $CH_{j}$, since the handover procedure must be ended before the node is out of range from $CH_{j}$. On the other hand, if MN moves at a slower speed, the threshold may be defined farther from $CH_{j}$, since there is more space to perform the handover procedure. Therefore, the mobile node speed must be considered for handover decision-making.

Consider that the MN moves toward the sink and back. Assuming that the entire mobility area is covered by the CHs and MN is associated to CH4, for instance, the worst case would occur when MN reaches the CH4 threshold ($th_{4}$), requests an association to CH3, changes its direction and returns. However, in the same way as above-mentioned, the handover procedure must be completed before MN reaches the next threshold, $th_{3}$ in this case. Therefore, in the proposed scenario, the direction of the node does not interfere in the association process.

### 3.2. Handover Mechanism

Considering the IEEE 802.15.4 PAN’s association and re-association mechanisms, we present some improvements considering multiple strategies. First, we use the FastA association of the IEEE 802.15.4e standard for the message exchange procedures. Secondly, we suppress the orphan scan step, as the proposed handover decision mechanism will be invoked before the link breakage. Thirdly, we reduce the number of channels in the scan operation, since the cluster-tree network is pre-set to operate in a single channel. Moreover, we propose the anticipation of the handover based on a proactive decision, and, finally, the adequate setup of the MAC layer parameters related to the association procedure is defined. Note that these improvements are backward compatible with IEEE 802.15.4 standard because they do not require any modification of the frames’ structure. Then, it is not expected to have any impact upon data processing in the devices.

The IEEE 802.15.4 standard does not specify a mechanism for the selection of an appropriate coordinator. However, the most commonly used parameter to trigger handover decisions is RSSI, as already shown in the state-of-the-art section. In beacon-enabled networks, the coordinator frequently sends a beacon to its child nodes. It allows nodes to synchronize with the parent coordinator and to exchange messages.

Thus, this work considers a handover decision approach based on the average of the RSSI values from the consecutive beacons received at the MN from each coordinator. This RSSI average from each CH is obtained using a sliding window technique, which prevents possible outliers of the RSSI average value due to distance, interference, and packet collisions.

**Figure 5:**
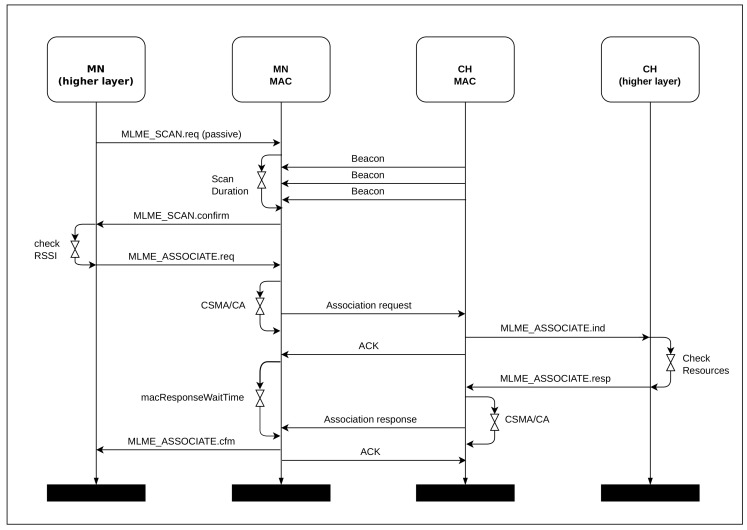
Message sequence chart for enhanced association mechanism.

Figure 5 shows the message exchange sequence for the enhanced association mechanism proposed in this work. This message exchange sequence improves the original association sequence defined in the IEEE 802.15.4 standard [2]. It illustrates that whenever a MN is activated in the network, it initiates the association process by listening for received beacons. When receiving beacons from a CH, the mobile node checks the beacon frame information, identifying cluster data, the active period, and RSSI value. Then, the MN prepares an association request and sends it to the candidate CH; then, it waits for an acknowledgment packet (ACK) and an association acknowledgment packet. The CH, upon receiving the join request of a mobile node, sends back an acknowledgment packet and evaluates whether there are adequate resources for the association. If the association is possible, the CH sends an association confirmation packet to the mobile node and the mobile node sends an acknowledgment packet to the CH. From this, the MN starts to send data packets during the active period of the associated CH.

According to this proposal, a mobile node should maintain a table with the RSSI values obtained from nearby CHs, which will be used for handover decision-making. Each MN associated with a specific CH will store the three most recent values of RSSI. Whenever moving, the mobile node will listen for beacons from other CHs, checking if the RSSI is higher than the established RSSI threshold. For these experiments, the threshold value was set at −87 dBm, which is considered to be an adequate threshold value [32]. If the average of the RSSI values of the received beacons is greater than this threshold, the MN checks if this value is greater than the average of the previously stored RSSI values. If this average is greater than the average RSSI values of the current CH, it requests an association with the new CH. If the association process is completed during the active period of the new CH, the MN synchronizes with the new CH and starts sending data packets in the active period of the new CH. Then, the CH generates a disassociation packet for the previous CH, which is sent during the active period. If the MN does not complete the association process, the MN will maintain the association with the previous CH.

The last improvement in the handover mechanism is related to the CSMA/CA default parameters of IEEE 802.15.4. In order to speed up message transmissions, this proposal uses alternative values for the *macMinBE* and *macMaxBE* parameters, as originally proposed in [33].

## 4. Simulation Assessment

This section presents a simulation assessment of the proposed enhanced association mechanism. The main objective is to compare the network behavior when applying the proposed mechanisms vs. the IEEE 802.15.4 standard mechanisms. In order to assess this proposal, the communication infrastructure was implemented in the OMNeT++ 4.6 (the OMNeT++ Simulator: https://omnetpp.org) network simulator with Castalia 3.3 framework. For this simulation, we have used the CT-SIM simulation model [34], which provides the required set of features to support a cluster-tree multi-hop topology.

### 4.1. Simulation Scenario

**Figure 6:**
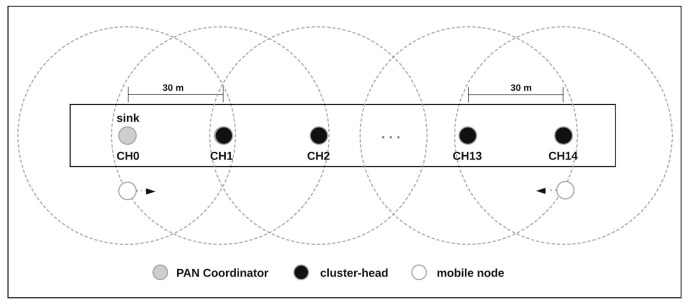
Communication scenario.

For the simulation, a communication scenario with a size of 450 m × 10 m was considered. The communication mechanism was implemented based upon a set of fixed nodes forming a backbone. This backbone was organized according to an in-line cluster-tree topology, where nodes acting as cluster-heads are deployed in-line to cover a pre-established monitoring area. Typical examples can be found in outdoor environments such as tunnels, roads or field athletics tracks, or indoor environments like a hospital hallway. Figure 6 illustrates these types of communication scenarios.

As shown in Figure 6, fifteen fixed nodes (CH0 to CH14) were positioned every 30 m simulating a typical athletics track, whose extension is 450 m. The CH0 node is the PAN coordinator, and node CH14 is the node positioned most deeply in the cluster-tree branch. The PAN coordinator is a special Full Function Device (FFD) acting as the root of the tree and as the sink node. The sink node acts as a gateway between the WSN and other networks (e.g., Internet). The deployment of CHs nodes guarantees that the coverage ranges of adjacent CHs are overlapped. Additionally, we consider that all CHs are connected to a power line supply and therefore, that they do not suffer from battery discharge problems. Finally, a set of mobile nodes (source-nodes) moving along the coverage area is also considered.

The number of mobile nodes varies from 1 to 40, and they are activated at different time intervals (offset), depending on the speed at which they move on the track. An initial offset time of 150 s was established for the overall network formation. The activation of the mobile nodes was defined considering that an MN only activates after the previous MN has moved 30 m. For example, if the mobile node number 1 moves at a speed of 1.4 m/s, the mobile node 2 will only be activated in the network in 171.4 s, that is, 21.4 s after the mobile node 1 has been activated, and so on. Mobile nodes are activated near to the sink node (CH0), move in the network coverage area until the end of the lane (CH14), and return uninterruptedly, allowing the association with the other CHs. The simulation ends when all packets from the mobile nodes are sent or the simulation reaches a predefined time limit.

The monitoring traffic is characterised by mobile nodes, and generate periodic messages at the same rate and send them to the PAN coordinator (sink) according to the communication rules defined by the IEEE 802.15.4 protocol. For the sake of simplicity, each mobile node generates a flow of 1000 messages to the sink node and the total simulation time was set to 50,000 s. For this simulation assessment, we defined two scenarios: mobile nodes moving at 1.4 m/s, emulating walking persons, and mobile nodes moving at 5 m/s, emulating running athletes. Table 1 summarizes the most important simulation configurations.

### 4.2. Results and Discussion

Simulations were performed comparing IEEE 802.15.4 standard association mechanisms (Std) with the improvements proposed in this paper, namely the anticipation of handover decision-making (Ant) and adequate setup of MAC parameters (MAC). In order to analyze network behavior, three metrics were defined. The average packet delivery rate is calculated as the ratio between the number of packets received by the PAN coordinator, and the number of packets generated by the mobile nodes. The average end-to-end communication delay represents the average communication delay of successfully received packets at the PAN coordinator, measured as the time interval from the start of its generation at the application layer of the mobile nodes till the time instant when it is received at the PAN coordinator. The average disconnected time percentage accounts for the average time that the mobile nodes remain disconnected from the network.

#### 4.2.1. Mobile Nodes at 1.4 m/s

**Figure 7:**
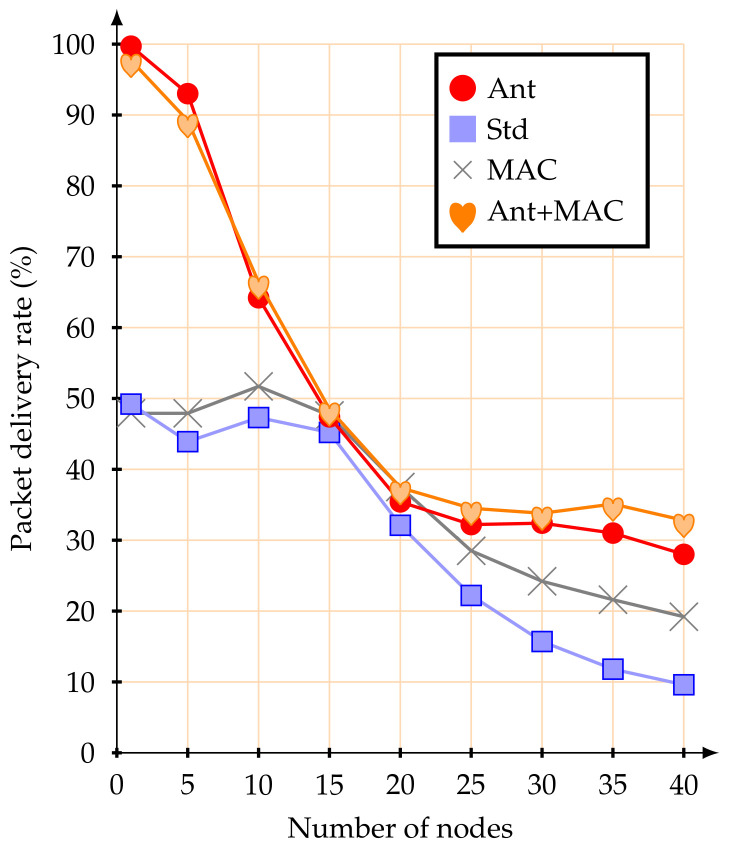
Average packet delivery rate at 1.4 m/s.

The first simulation experiments were performed with a set of mobile nodes (from 1 to 40) moving at 1.4 m/s. Figure 7 illustrates the average packet delivery rate.

Using the standard association parameters, the average packet delivery rate was below 50%, even for a small number of active mobile nodes. This value remains more or less constant up to 15 mobile nodes, from this point the average packet delivery rate is considerably reduced, until the network became fully saturated. This behavior was already expected due to the CSMA-CA contention mechanism. These results, namely the reduction of the packet delivery rate to 50%, demonstrate the inefficiency of the association mechanism of the IEEE 802.15.4 standard when using mobile nodes with multi-hop topology networks. If alternative MAC parameters are used, there was no significant improvement in the packet delivery rate for a small number of mobile nodes. However, as the number of mobile nodes increases, the influence of managing the MAC parameters becomes visible.

When configuring the network with the proposed improvements (anticipation of handover decision-making), the results were significantly improved. With up to five mobile nodes, the average packet delivery rate was greater than 90%. Similar values were obtained when in conjunction with the alternative MAC parameter strategy. The proposed scheme is clearly more efficient than the IEEE 802.15.4 standard basic association mechanism, for a network with up to 10 mobile nodes.

**Figure 8:**
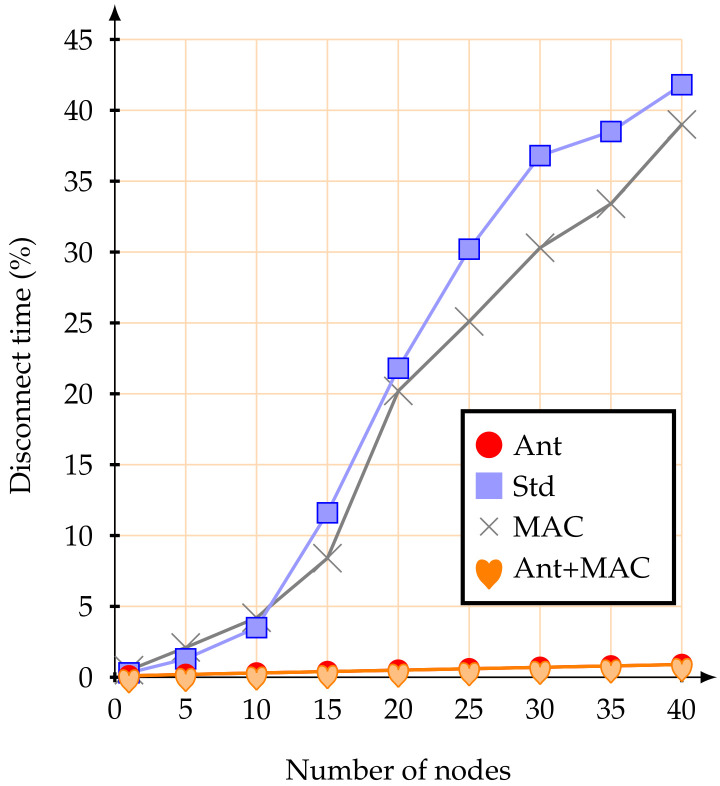
Average percentage of disconnect time at 1.4 m/s.

Regarding the percentage of time during which the mobile nodes remain disconnected from the network (blackout interval), the use of the proposed anticipation mechanism clearly improves the behaviour of the IEEE 802.15.4 standard association mechanisms. Figure 8 illustrates the average percentage of time during which the mobile nodes remain disconnected from the network.

The achieved results highlight that the proposed association mechanism allows mobile nodes to remain synchronized with the network nearly all the time. This behaviour is possible because the mobile nodes are always verifying the RSSI signal from the beacons received from the CHs. Therefore, a mobile node losing synchronization with the current CH, if it verifies that its RSSI signal has gone below the pre-established threshold and there is another CH with a better signal, it will immediately request an association to the new CH (make-before-break strategy). If the new association is not successful, the mobile node remains associated with the current CH. This effectively prevents mobile nodes from losing synchronization with the network, allowing them to send their messages during the active periods of their parent nodes (CHs). Even when facing a considerable increase in the number of mobile nodes, the proposed methodology is better. When using the IEEE 802.15.4 standard association mechanism, if the number of mobile nodes increases, the average time during which the nodes remain disconnected from the network clearly also increases. For example, with 40 mobile nodes, the average percentage of time during which the nodes remained disconnect from the network was 41.8%—that is, they did not have access to the network for almost half of the active time. When just adjustment of MAC parameters was used, the synchronization with the network was similar. Therefore, the reason why the mobile nodes kept synchronized with the network was the anticipation method of the handover decision.

**Figure 9:**
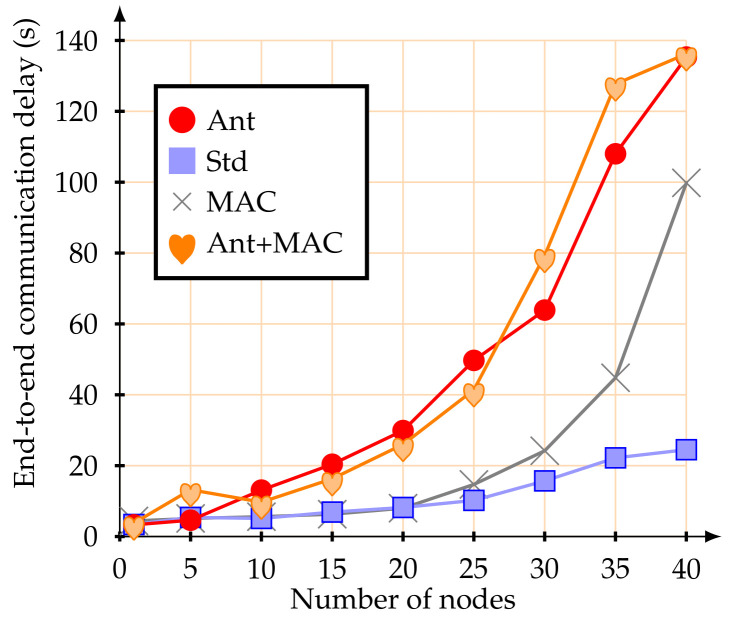
Average end-to-end delay at 1.4 m/s.

Figure 9 illustrates the average end-to-end communication delay. It is important to remark that this metric can be affected by several factors, such as: wireless channel access mechanism, number of contention nodes, number of hops to sink node, and the average size of the queues (buffer size). Besides, for this work, all clusters have the same allocated active periods, i.e., it is not used any mechanism to proportionally allocate superframe duration size for CHs, as recently proposed in [35].

As it can be observed in Figure 9, when using the proposed anticipation mechanism with a few active mobile nodes, the average end-to-end communication delay was very similar to the IEEE 802.15.4 standard mechanism. As the number of mobile nodes increased, it also increased this communication delay. In what concerns the IEEE 802.15.4 standard, the end-to-end communication delay seems to remain more or less constant up to 25 mobile nodes. Nevertheless, it cannot be underestimated the fact that, for a number of mobile nodes larger than 10, most parts of these nodes became disconnected from the network (Figure 8) and the percentage of packets that are lost is larger than 35% (Figure 7). Therefore, as only packets that are successfully delivered to the PAN coordinator are considered for the end-to-end delay average evaluation, the standard end-to-end delay, although lower, must be interpreted together with the packet delivery rate.

In fact, as the proposed anticipation mechanism guarantees a higher packet delivery rate (Figure 7), there is a higher number of data packets travelling in the cluster-tree network towards the PAN coordinator. Considering that this work does not implement any optimized superframe duration allocation mechanism, data messages may experience greater end-to-end communication delays mainly due to the delay of the buffer queues and network congestion.

#### 4.2.2. Mobile Nodes at 5 m/s

**Figure 10:**
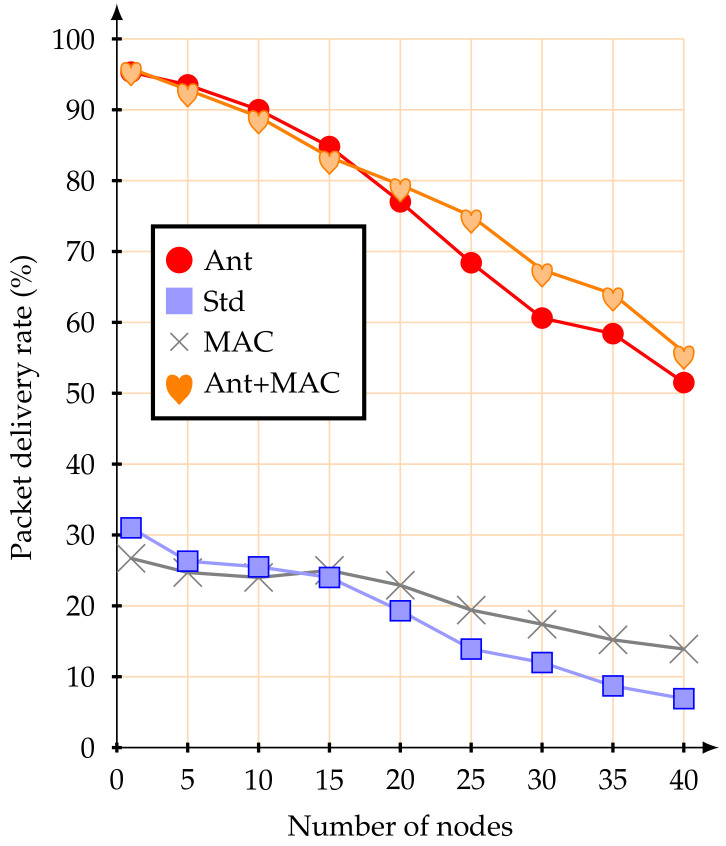
Average packet delivery rate at 5 m/s.

In the same way, as in the previous scenario, a new set of tests was performed, but with mobile nodes moving at 5 m/s. Figure 10 shows the related average packet delivery rate.

The results show that using the IEEE 802.15.4 standard association mechanisms, the packet delivery rate was below 31% with just one mobile node, while using the proposed anticipation method, it reached more than 90% of packet delivery rate. It can also be observed that with the proposed anticipation method and up to 40 active mobile nodes, the average packet delivery rate was always over 55%. The use of alternative MAC parameters did not make a significant difference in the packet delivery rate for a small number of mobile nodes. It was, however, possible to identify that this method, together with the anticipation mechanism, presents better results for a large number of mobile nodes. On the other hand, it is possible to conclude that, with an increasing speed, the IEEE 802.15.4 standard association mechanisms were highly inefficient to support mobile nodes.

**Figure 11:**
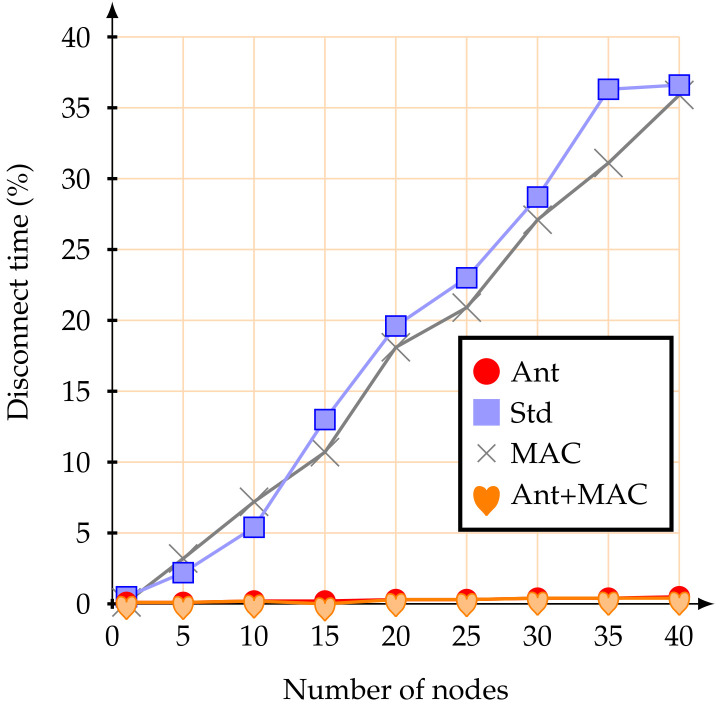
Average percentage of disconnect time at 5 m/s.

Regarding the time during which the mobile nodes remained disconnect from the network, the results were very similar to the first scenario, with mobile nodes at 1.4 m/s. Figure 11 illustrates the average percentage time during which the mobile nodes remained disconnect from the network.

Using the standard association mechanism, the simulation results showed that with 10 or more mobile nodes, there is a large increase in the percentage during which the nodes were without access to the network. On the other hand, with the proposed anticipation method, the mobile nodes rarely lose synchronization with the network. It is possible to conclude that, with the proposed anticipation method and for the presented scenario, even increasing the node speeds and the number of mobile nodes, the mechanism maintained the synchronization of the nodes with the network.

**Figure 12:**
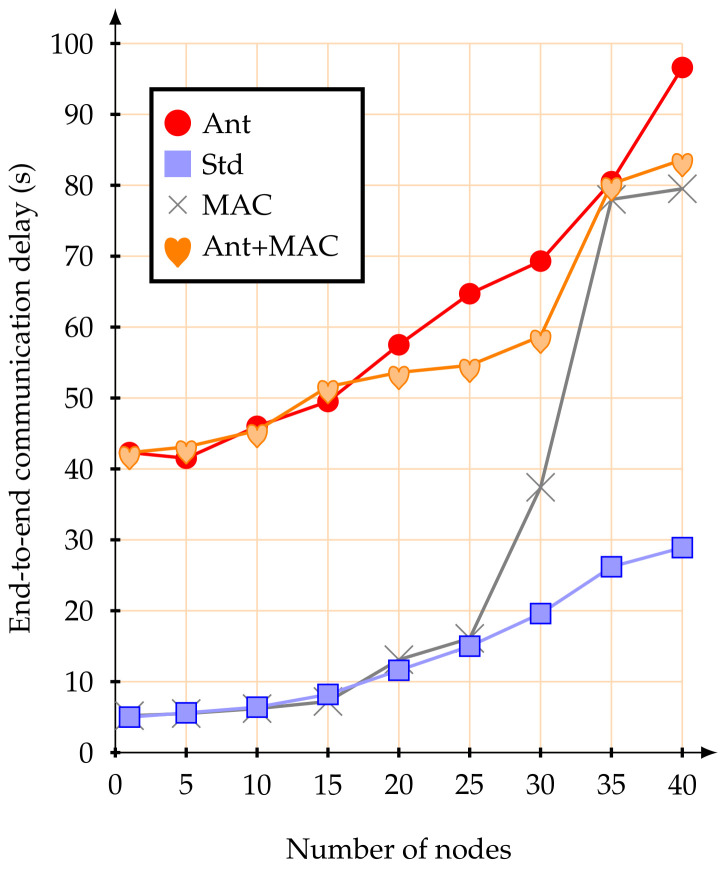
Average end-to-end delay at 5 m/s.

Finally, Figure 12 illustrates the end-to-end communication delays for mobile nodes at 5 m/s. Results are similar to the previous scenario, which show that a higher number of travelling messages implies higher end-to-end delays due to network congestion. It can be attributed to the evident gain in the packet delivery rates, even when the number of mobile nodes increases (Figure 10).

## 5. Conclusions

This paper proposes a new association and re-association scheme to handle mobile nodes in IEEE 802.15.4 cluster-tree networks. We first presented the state-of-the-art on handover mechanisms for WSNs through a systematic literature review. Next, a proposal for the improvement of the association mechanisms was systematized using a set of strategies. We also assessed the protocol constraints for network formation, such as BI and beacon scheduling and superframe duration allocation. The communication infrastructure was validated via extensive simulation tests, exploring the proposed improvements. It was possible to prove the efficiency of the proposed anticipation method, in relation to packet delivery rates as maintaining the connectivity of the mobile nodes with the network. Simulation results show that it is possible to improve the IEEE 802.15.4 standard association mechanisms, to support wide-scale WSN applications with mobile nodes. The following key conclusions can be summarized:

- Current WSN standards do not efficiently support node mobility, especially if they are being used to support critical applications with real-time and reliability requirements;
- Currently, available Wireless Body Area Network (WBAN) / WSN technologies and standards do not support high packet delivery rates when multiple hop topologies are used;
- Patient mobility in health monitoring applications such as physiotherapy requires high packet delivery rates combined with small and predictable end-to-end communication delays. Therefore, it is essential to ensure efficient handover mechanisms so that patients do not lose connection from the network while moving around the coverage area;
- This paper has shown that by using a combination of strategies, it is possible to enhance the IEEE 802.15.4 standard association mechanisms to support node mobility and, therefore, to meet the health monitoring applications requirements.

In summary, results reported in this paper represent a step forward when compared to the IEEE 802.15.4 standard association mechanisms, guaranteeing that mobile nodes remain connected during longer periods of time with coordinator nodes. As future work, we intend to integrate an optimized active period allocation mechanism for cluster-tree networks and to evaluate the proposed mechanism by experimental analysis, comparing the power consumption and other metrics.

## Figures and Tables

**Table 1:** Simulation configuration.

Definition	Standard Value
Radio	cc2420
Frequency	2.4 ghz
Environment	450 m × 10 m
Packet rate	4 pkt/s
Number of mobile nodes	1 to 40
Simulation time	50,000 s
Packets per node	1000
Beacon Order	8
Superframe Order	2

## Abbreviations

The following abbreviations are used in this manuscript:

AP	Access Point
BAN	Body Area Network
BI	Beacon Interval
BO	Beacon Order
CH	Cluster-head
CFP	Contention-Free Period
CSMA-CA	Carrier Sense Multiple Access with Collision Avoidance
CW	Contention Window
FAM	Fast Association Mechanism
FFD	Full Function device
FQDA	Fuzzy Quantitative Decision Algorithm
IEEE	Institute of Electrical and Electronics Engineers
IoMT	Internet of Medical Things
LQI	Link Quality Indicator
MAC	Media Access Control
MN	Mobile Node
PAN	Personal Area Network
RSSI	Received Signal Strength Indication
SAP	Simple Association Process
SD	Superframe Duration
WBAN	Wireless Body Area Network
WSN	Wireless Sensor Network
